# Society Meetings

**Published:** 1894-09

**Authors:** 


### Society Meetings.

THE American Association of Obstetricians and Gynecologists will hold its seventh annual meeting at Toronto, Ont., Wednesday, Thursday and Friday, September 19, 20 and 21, 1894.

The following corrections in the program published last month are announced: (18) The Present Status of the Surgical Treatment of Uterine Fibroids, L. S. McMurtry, Louisville, Ky.; (27) Restoration of Intestinal Continuity Without Mechanical Devices, William E. B. Davis, Birmingham, Ala. The following additions have been made: (29) Chronic Atrophy of the Vulva (Kaurosis Vulvæ), Its Pathology and Radical Treatment, Charles A. L. Reed, Cincinnati, O.; (30) The Reason Why Patients Recover from Tuberculosis of the Peritoneum After Operation, Robert T. Morris, New York; (31) Report of Two Cases of Injury of the Ureter following Operation for Cancer of the Uterus, L. H. Laidley, St. Louis, Mo.; (32) Vaginal Fixation of the Uterus as a Cure for Retrodisplacements, Clinton Cushing, San Francisco, Cal.; (33) Hydrosalpinx, A. H. Cordier, Kansas City, Mo. (34) *Discussion*—Should Antiseptic Vaginal Douching be made a Routine Practice in the Puerperium? Referees—A. H. Wright, Toronto, Thos. Lothrop, Buffalo, J. Edwin Michael, Baltimore, Md., H. T. Machell, Toronto; (35) Infectious Diseases During Pregnancy, A. H. Wright, Toronto; (36) Congenital Diaphragmatic Hernia—Reports of Two Cases, H. T. Machell, Toronto; (37) Report of Some Interesting Abdominal Operations, with Exhibition of Specimens, Rufus B. Hall, Cincinnati, O.; (38) A New Operation for the Radical Cure of Inguinal and Femoral Hernia, Charles A. L. Reed, Cincinnati, O.; (39) *a*, Present Status of Treatment of Pelvic Inflammations; (*b*) A Question of Priority, Walter B. Dorsett, St. Louis, Mo.; (40) Uterine Malposition and Disease as a Cause of Insanity, George R. Shepherd, Hartford, Conn. (41) *a*, Appendicitis, Four Cases Surgically Treated in thirty-seven consecutive hours; (*b*) Supplementary Paper on Ectopic Pregnancy, George S. Peck, Youngstown, O.

---

The Association of American Medical Editors elected officers for the ensuing year as follows: President, John B. Hamilton, of Chicago; vice-president, G. M. Gould, of Philadelphia; secretary, H. B. Ellis, of San Francisco. The next meeting will be held at Baltimore during the session of the American Medical Association at that city.

---

The Medical Association of Central and Western New York will hold its next annual meeting in Buffalo, Tuesday, October 16, 1894.

